# Toward a cell-free hydantoinase process: screening for expression optimization and one-step purification as well as immobilization of hydantoinase and carbamoylase

**DOI:** 10.1186/s13568-017-0420-3

**Published:** 2017-06-09

**Authors:** Christin Slomka, Georg Paris Späth, Phillip Lemke, Marc Skoupi, Christof M. Niemeyer, Christoph Syldatk, Jens Rudat

**Affiliations:** 1 Institute of Process Engineering in Life Sciences, Section II: Technical Biology, Karlsruhe Institute of Technology (KIT), Engler-Bunte-Ring 3, 76131 Karlsruhe, Germany; 2 Institute of Biological Interfaces (IBG-1), Karlsruhe Institute of Technology (KIT), Hermann-von-Helmholtz-Platz 1, 76344 Eggenstein-Leopoldshafen, Germany

**Keywords:** Amino acids, Enzyme catalysis, Hydantoinase process, Protein purification, Enzyme immobilization, Magnetic beads

## Abstract

The hydantoinase process is applied for the industrial synthesis of optically pure amino acids via whole cell biocatalysis, providing a simple and well-established method to obtain the catalyst. Nevertheless, whole cell approaches also bear disadvantages like intracellular degradation reactions, transport limitations as well as low substrate solubility. In this work the hydantoinase and carbamoylase from *Arthrobacter crystallopoietes* DSM 20117 were investigated with respect to their applicability in a cell-free hydantoinase process. Both enzymes were heterologously expressed in *Escherichia coli* BL21DE3. Cultivation and induction of the hydantoinase under oxygen deficiency resulted in markedly higher specific activities and a further increase in expression was achieved by codon-optimization. Further expression conditions of the hydantoinase were tested using the microbioreactor system BioLector^®^, which showed a positive effect upon the addition of 3% ethanol to the cultivation medium. Additionally, the hydantoinase and carbamoylase were successfully purified by immobilized metal ion affinity using Ni Sepharose beads as well as by functionalized magnetic beads, while the latter method was clearly more effective with respect to recovery and purification factor. Immobilization of both enzymes via functionalized magnetic beads directly from the crude cell extract was successful and resulted in specific activities that turned out to be much higher than those of the purified free enzymes.

**Electronic supplementary material:**

The online version of this article (doi:10.1186/s13568-017-0420-3) contains supplementary material, which is available to authorized users.

## Introduction

**Fig. 1:**
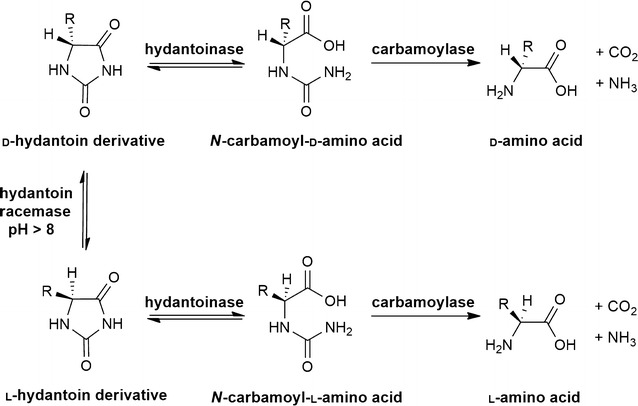
Hydantoinase process for the synthesis of optically pure α-amino acids starting from racemic 5-monosubstituted hydantoins [Modified after Syldatk et al. (1999)]

The hydantoinase process (Fig. 1) is well established in industry for the biocatalytic production of enantiopure *α*-amino acids, especially *α*-d-amino acids like *α*-d-phenylglycine and *α*-d-*p*-hydroxyphenylglycine. They serve as side chains of the semisynthetic antibiotics ampicillin and amoxicillin (May et al. 2000; Bommarius et al. 2001). Since racemization is enabled by hydantoin racemases or spontaneous racemization of unreacted substrates under slightly alkaline conditions (Ware 1950; Kato et al. 1987; Las Heras-Vazquez et al. 2009), this process allows a dynamic kinetic resolution and therefore a maximum yield of 100%.

To date, the use of whole cell biocatalysis is widespread in industrial biocatalysis due to the easy access to the biocatalyst, low production costs and simple separation of biocatalyst and product. Nevertheless, there are also some drawbacks using whole cell biocatalysis like transport limitations of substrates as well as intermediates and products. Furthermore, uncontrollable degradative side reactions may take place inside of the cells and especially for the hydantoinase process, the substrate solubility is relatively low (Slomka et al. 2014). Therefore, cell-free reaction systems came into focus.

The application of recombinantly expressed enzymes using well-established expression hosts like *Escherichia coli* allows an overexpression of the target enzyme as well as the attachment of tags for purification and immobilization (Chen 2012). Regardless, the varying codon-usage from the different species may lead to premature termination of translation, expression of non-functional or insoluble proteins or a complete lack of expression. This points out that codon-optimization of the desired gene according to the host organism, for instance, is an effective tool to improve the expression and therefore avoid misfolding and aggregation of the protein (Kurland and Gallant 1996).

Furthermore, it should be noted that the use of affinity tags for purification of proteins may also interfere with protein folding, depending on their location and amino acid composition (Terpe 2003). Although several cases are reported in which inclusion bodies have been used for industrial scale protein refolding, they often represent rather inactive enzyme aggregates; thus their formation should be avoided. In addition to changes in cultivation as well as induction conditions to slow down the expression and allow correct folding of the enzymes, the utilization of chaperones got into focus. They either can be induced by causing physical stress or coexpressed to support the correct folding of enzymes (Thomas and Baneyx 1996; Marco et al. 2007).

Since the performance of whole cell biotransformations involves disadvantages like for instance transport limitations and low substrate solubility in aqueous systems, the isolation and purification of recombinantly expressed proteins is often advantageous. To name only some examples of the available toolbox, there is e.g. the possibility to use calmodulin-binding peptides, cellulose-binding domains, maltose-binding proteins, FLAG-tags, Arg-tags, His-tags, Strep-tags and SBP-tags (Terpe 2003). In addition to purification, the immobilization of enzymes is a suitable method for an easier handling and controllability as well as for stabilization and reutilization (Swartz 2011). Concerning hydantoinases and *N*-carbamoylases, different immobilization methods have been developed using various supports like DEAE-cellulose, polyacrylamide, activated charcoal and calcium alginate (Yamada et al. 1980; Meyer and Runser 1993; Lee et al. 1996; Foster et al. 2003). Additionally, immobilization by coupling to Eupergit C and C 250 L by oxirane groups as well as by amino groups was performed successfully for both enzymes (Ragnitz et al. 2001a; Bulawayo et al. 2006). The use of different matrices together with different surface modifications is often time-consuming and inefficient in practice, plus immobilization by covalent bond reagents may result in deactivation of the enzymes (Ko et al. 2012). Since a metal-affinity based approach allows integrated purification and immobilization of enzymes in a simple procedure, it got into focus lately. Ho et al. (2004) applied silica-based IMAC adsorbents for the purification and immobilization of the d-hydantoinase from *Bacillus caldolyticus*. Later, the simultaneous purification and immobilization of a d-hydantoinase was enabled by using an immobilized metal affinity membrane. The membrane-immobilized enzyme showed higher pH and temperature tolerances and was reusable for 15 times with 99% of its original activity (Ko et al. 2012, 2013).

In this work, the expression of the hydantoinase from *Arthrobacter crystallopoietes* DSM 20117 was optimized by codon-optimization and furthermore, a screening for the indirect induction of chaperones as well as for coexpression of different chaperones was carried out to improve soluble hydantoinase expression. Taking this recombinantly expressed hydantoinase as well as the carbamoylase from *A. crystallopoietes* DSM 20117, the purification by affinity chromatography (IMAC) was established for both enzymes. Furthermore, immobilization of both enzymes was investigated via metal ion affinity using functionalized magnetic beads, allowing a facilitated process control as well as an easy removal of the catalysts.

## Materials and methods

Chemicals were of reagent grade and obtained from commercial sources if not stated otherwise. Phenylhydantoin (PheHyd), benzylhydantoin (BnH) and hydroxymethylhydantoin (HMH) were synthesized according to Stark and Smyth (1963). The corresponding *N*-carbamoylamino acids *N*-carbamoyl-α-phenylglycine (*N*CPheGly), *N*-carbamoyl-α-phenylalanine (*N*CPheAla) and *N*-carbamoyl-α-serine (*N*CSer) were synthesized after Suzuki et al. (1973).

### Buffers and solutions

#### Purification via Ni Sepharose high performance beads (GE Healthcare, UK)

The wash- and binding buffer was 50 mM Tris–HCl (500 mM NaCl, 20 mM imidazole, pH 8), while for purification of the carbamoylase, 5 mM DTT as reducing agent were added. As elution buffer, 50 mM Tris–HCl (500 mM NaCl, 500 mM imidazole, pH 8) was used. For purification of the hydantoinase, the buffer was supplemented with 1 mM ZnSO_4_, while for the carbamoylase 5 mM DTT were added.

#### Purification and immobilization via Dynabeads^®^ His-tag isolation and pulldown (Life Technologies, Norway)

As wash- and binding buffer, 50 mM NaPP buffer (300 mM NaCl, 0.01% TWEEN^®^ 20, pH 8) was used, while the elution buffer additionally contained 300 mM imidazole. For functionalization of the magnetic beads with cobalt, 10 mM CoCl_2_ in _dd_H_2_O was used.

*Buffer exchange* of samples containing imidazole after elution is crucial for the activity of enzymes. Furthermore, since the functionalized magnetic beads are not compatible with DTT, after purification of the carbamoylase DTT was supplied subsequently by buffer exchange. For this purpose, Slide-A-Lyzer™ MINI Dialysis Devices (Thermofisher Scientific, US) were used and applied according to manufacturer´s instructions.

*Catalysis buffer* for biotransformation assays was 50 mM Tris–HCl (pH 8.0) in each case. Additionally, for hydantoinase approaches, a final concentration of 1 mM ZnSO_4_ was added while for carbamoylase approaches 5 mM DTT were added as reducing agent. Substrate as well as product solutions were prepared in catalysis buffer.

*Ehrlich’s reagent* for photometric analysis of *N*-carbamoylamino acids was 1 g dimethylaminobenzaldehyde in 5 mL conc. HCl and 5 mL _dd_H_2_O (Werner 2001).

### Media

The medium for cultivation and induction of the *E. coli* BL21DE3 harboring the plasmid for the hydantoinase was terrific broth (TB) medium containing 12 g/L tryptone, 24 g/L yeast extract, 0.4% v/v glycerol (99.5%), 17 mM potassium dihydrogen phosphate and 7 mM di-potassium hydrogen phosphate. For cultivation and induction of the *E. coli* BL21DE3 harboring the plasmid for the carbamoylase was lysogeny broth (LB) medium containing 10 g/L tryptone, 5 g/L yeast extract and 10 g/L NaCl. The pH was adjusted to 7 with NaOH.

### Bacterial strains and plasmids

*Escherichia coli* BL21DE3 harboring the plasmid pDEST42 was used for recombinant expression of the hydantoinase from *A. crystallopoietes* DSM 20117 with *C*-terminal His-tag (d-Hyd). *E. coli* BL21DE3 harboring the plasmid pET28a was used for expression of the codon-optimized hydantoinase from *A. crystallopoietes* DSM 20117 with *C*-terminal His-tag and *N*-terminal SBP-tag [d-Hyd(co)]. For expression of the carbamoylase from *A. crystallopoietes* DSM 20117 with *C*-terminal His-tag (d-Carb), *E. coli* BW 3110 containing the plasmid pMW1 was used (Werner et al. 2004).

### Codon-optimization

To prevent the expression of non-functional and insoluble proteins, the d-hydantoinase from *A. crystallopoietes* DSM20117 was codon-optimized and in addition to the *C*-terminal His-tag a *N*-terminal SBP-tag was added [d-Hyd(co)]. The gene encoding the d-hydantoinase from *A. crystallopoietes* DSM 20117 was optimized based on the codon bias of *E. coli* for improved soluble expression using the GeneArt™ software.

### Chaperone sets (Takara Bio Inc.)

For coexpression of the the codon-optimized hydantoinase from *A. crystallopoietes* DSM 20117 with chaperones, the following chaperone sets were used: C1—dnaK-dnaJ-grpE, groES-groEL (pG-KJE8); C2—groES-groEL (pGro7); C3—dnaK-dnaJ-grpE (pKJE7); C4—groES-groEL-tig (pG-Tf2); C5—tig (pTf16).

### Cultivation and induction

The preculture for every cultivation was prepared in 100 mL baffled shaking flasks by adding 20 mL medium, 10 μL of a glycerol stock as well as the required antibiotic for selection. Cultivation of the precultures was conducted at 37 °C and 120 rpm for 18 h.

For expression of the hydantoinase, *E. coli* BL21DE3 harboring the plasmid pDEST42 or pET28a was cultivated in TB-medium (3% v/v EtOH). For inoculation, precultures were added to 200 mL medium and 100 µg/mL ampicillin (pDEST42) or 100 µg/mL kanamycin (pET28a) in 1 L baffled shaking flasks to an OD_600_ of 0.2. The cells were cultivated at 37 °C and 120 rpm and induction was carried out at an OD_600_ of 4. Subsequently, the cultivation was continued to an overall cultivation time of 24 h, starting from inoculation. For harvesting, after 15 min of centrifugation at 4700×*g* and 4 °C, the cells were quick-frozen with liquid nitrogen and stored at −20 °C until cell disruption and purification or immobilization.

Cultivations for the screening of expression conditions were carried out under the same conditions as described above, but in 48-well Flower-Plates^®^ (m2p-labs, Germany) and in a culture volume of 1 mL. For continuous control of growth, a BioLector^®^ MB microfermentation system (m2p-labs, Germany) was used as an incubator (37 °C, 600 rpm, gain of light scattering: 20, humidity: 95%, measuring interval: 10 min, cultivation time: 20 h). The induction times were calculated based on scattered light values in the BioLector^®^ system using previously determined OD_600_/scattered light correlation functions. For 600 rpm, the function y(OD_600_) = 0.0794 × x (scattered light) − 1.4317 was determined. Cultivation setups for the screening were cultivation at pH 6 and 7, ±3% EtOH and ±1 mM IPTG. The activities of d-Hyd and d-Hyd(co) were tested at every combination of conditions. Furthermore, for d-Hyd(co), coexpression of five different chaperone sets (C1–C5) was tested at different cultivation setups (±EtOH, ±IPTG), resulting in a total of 28 different cultivation conditions for d-Hyd(co) as well as 8 different cultivation conditions for d-Hyd.

The cultivations were carried out each for three times in triplicates, using two cultures of the triplicate for whole cell biotransformation assays and the third culture for mechanical cell disruption by sonication and subsequent SDS-PAGE analysis as well as for determination of the cell dry weight. This finally results in triplicates for every cultivation setup.

For expression of the d-Carb, *E. coli* BW 3110 harboring the plasmid pMW1 was cultivated in LB-medium (Werner et al. 2004). 2 L baffled shaking flasks were used for 400 mL of culture volume. Inoculation was carried out by adding the preculture to obtain an OD_600_ of 0.1 and subsequently cultivation was carried out at 37 °C and 120 rpm up to an OD_600_ of 0.4–0.6. Then, protein expression was induced by adding rhamnose to a final concentration of 2 mg/mL and cultivation was continued at 30 °C and 120 rpm for 6 h. For harvesting, after 15 min of centrifugation at 4700×*g* and 4 °C, the cells were quick-frozen with liquid nitrogen and stored at −20 °C.

### Cell disruption

For every cell pellet except from BioLector^®^ cultivations, chemical cell disruption was carried out using Bug Buster^®^ (Merck Novagen, US) according to manufacturer’s instructions. After 75 min centrifugation at 4 °C and 4700 rpm to separate the crude extract from cell debris and insoluble proteins, 10 μL of the protease inhibitor was added per 1 mL of crude extract which was cooled permanently and directly used for further investigations. The insoluble part resulting from cell disruption was stored at −20 °C until SDS-PAGE analysis.

In cultures from BioLector^®^ cultivations, the cells were mechanically disrupted by sonication with an 8-tip sonication probe for SDS-PAGE analysis. For this purpose, one culture of a triplicate was centrifuged for 10 min at 12,000 rpm and the supernatant was discarded. After washing for three times with Tris–HCl buffer (50 mM, pH 8), the resulting cell pellet was resuspended in 900 μL _dd_H_2_O. 150 μL of this cell suspension was transferred to a 96-well microtiter plate to perform sonication with an amplitude of 70%. After each 20 s of sonication, an interruption of 30 s followed. This sequence was repeated for six times and the 96-well microtiter plate was cooled permanently. For separation of the crude cell extract from cell debris, the plate was centrifuged for 90 min at 4000 rpm and 4 °C. The resulting insoluble fraction was resuspended in 300 μL _dd_H_2_O and both the crude extract and the insoluble fraction were stored at −20 °C for further analysis.

### Purification and immobilization of enzymes

Both enzymes were purified using two different methods to compare their efficiency. The first method was purification using Ni Sepharose high performance beads in a batch approach. Initially, 2 mL of the Ni Sepharose high performance stock solution (stored in 20% ethanol) were taken and washed several times. For the first washing step, the Ni Sepharose beads were resuspended in 5 mL _dd_H_2_O and subsequently centrifuged for 1 min at 4 °C and 4700 rpm. Then three more washing steps were carried out with 5 mL wash- and binding buffer supplied with additives for the particular enzyme. For every purification approach, 1 mL of this prepared bead suspension was incubated with 4 mL crude cell extract for 60 min in an overhead shaker at 4 °C. Preliminary tests showed, that in contrast to the standard protocol of GE Healthcare, where two times washing and resuspending in wash-and binding buffer are recommended, three washing steps with 1 mL wash-and binding buffer are necessary to avoid non-specific binding of proteins. Furthermore, an elution gradient was applied beginning with an elution step (eluate 1) using 2 mL of a 25% v/v elution buffer (125 mM imidazole) and 15 min incubation in an overhead shaker at 4 °C. After 1 min centrifugation at 4700 rpm and 4 °C and collection of the supernatant, a second elution step (eluate 2) like this was carried out with 100% v/v elution buffer (500 mM imidazole). The supernatants of every washing- and elution step were collected and cooled for further investigations.

Another method for the purification of d-Hyd(co) as well as d-Carb was tested by using Dynabeads^**®**^ His-tag Isolation and Pulldown. To prevent reduction of the coordinating cobalt ions, reducing agents like DTT need to be avoided. For every approach, 20 mg/mL functionalized magnetic beads were used, fractionated in 2 mL micro test tubes. Since their storage occurs in 20% ethanol, first of all the magnetic beads were washed for four times with 0.5 mL wash- and binding buffer by using a magnetic device for removal of the supernatant. To this 20 mg/mL magnetic beads, 1 mL of crude extract was added and this mixture was incubated for 5 min at 25 °C and 800 rpm. For purification of the d-Carb, a final concentration of 12 mM imidazole was added to the crude extract for avoiding non-specific binding of proteins containing histidines. This was not the case for purification of the hydantoinase, since imidazole would interfere with the zinc ions that are crucial for its activity. Then, the supernatant was collected and cooled at 4 °C for later analysis. The loaded magnetic beads were washed for four times with 0.5 mL wash- and binding buffer. For elution of each enzyme, 0.5 mL elution buffer was added, followed by incubation at 25 °C and 800 rpm for 5 min. The resulting eluate was collected by using a magnetic device and cooled at 4 °C. For functionalization and recyclation of used magnetic beads, they were washed for three times with elution buffer and subsequently with a 10 mM CoCl_2_ solution.

Buffer exchange of samples containing imidazole after elution is crucial for the activity of enzymes and was carried out using Slide-A-Lyzer™ MINI Dialysis Devices (Thermofisher Scientific, US) and catalysis buffer.

Immobilization of the enzymes via His-tag applying Dynabeads^®^ His-tag isolation and pulldown was carried out as described above, but without the elution step. The loaded and washed magnetic beads were resuspended in 0.5 mL of the particular catalysis buffer to perform the biotransformation.

### Assays of enzyme activity

#### Whole cell biotransformation assays

For whole cell biotransformation assays concerning expression optimization of the hydantoinase, the harvested cells were thawed on ice, washed twice with catalysis buffer and resuspended in the same buffer in a ratio of 10 mL buffer for 100 mL of harvested culture. 750 μL from this cell suspension were added to 750 μL of substrate solution (4 mM in catalysis buffer) to start the biotransformation reaction. Negative controls were performed on the one hand by adding catalysis buffer instead of cell suspension and on the other hand by adding catalysis buffer instead of the substrate. The assay was carried out at 40 °C at 800 rpm and samples were taken at selected reaction times by withdrawing 200 μL from the reaction mixture, centrifugation at 13,000 rpm for 5 min and storage of the supernatant at −20 °C until analysis. For determination of cell dry weight (cdw), micro reaction tubes were dried overnight at 60 °C and subsequently weighed (in triplicates). After adding 1 mL culture volume and centrifugation for 5 min at 13,000 rpm, the supernatant was discarded and they were dried overnight at 60 °C again to determine the cell dry weight.

Whole cell biotransformation assays of the BioLector^®^ cultures were performed in a smaller scale, because less culture volume was obtained. Two cultures of the triplicates were united in one micro reaction tube, centrifuged for 10 min at 12,000 rpm and the supernatant was discarded. The cell pellets were washed twice with 500 μL catalysis buffer and then resuspended in 200 μL of the same buffer. By adding 200 μL of a 4 mM solution of phenylhydantoin, the reaction started and was conducted at 40 °C and 800 rpm. At selected reaction times, 20 μL samples were taken and after centrifugation at 13,000 rpm for 5 min, the supernatant was collected and stored at −20 °C for further analysis.

#### Biotransformation assays using crude cell extract or purified enzymes

After chemical cell disruption, the protein concentration of crude cell extract was determined using the Protein Quantification Kit BCA-Assay (Interchim, France) according to manufacturer´s instructions. Then the crude cell extract was diluted to 10 mg/mL with catalysis buffer. The samples received from enzyme purification and subsequent buffer exchanges were used without prior dilution, but protein concentration was determined to calculate enzyme activities. To start the biotransformation reaction, the prepared crude cell extract or purified enzyme was added to the substrate solution (10 mM in catalysis buffer) in a ratio of 1:1. Additionally, negative controls were conducted by adding catalysis buffer instead of crude cell extract/purified enzyme or instead of substrate. Hydantoinase activity was determined at 40 °C and 800 rpm, whereas assays employing d-Carb were carried out at 30 °C and 800 rpm. At selected reaction times, 100 μL samples were taken and treated differently, depending on the following analytical method. When HPLC-analysis was conducted, the reactions were stopped by 10 min incubation of the samples at 95 °C and subsequent centrifugation for 5 min at 13,000 rpm. The supernatant was collected and stored at −20 °C until analysis. For photometric analysis applying Ehrlich’s Reagent, the samples were added to an equal amount of conc. HCl. After resuspending, the samples were centrifuged for 5 min at 13,000 rpm and the supernatant was also stored at −20 °C until analysis.

#### Biotransformation assays using immobilized enzymes

Magnetic beads carrying His-tagged enzymes were resuspended in 500 μL catalysis buffer to conduct the biotransformation assay. By adding 500 μL of the particular substrate solution (10 mM in catalysis buffer), the reaction was started. As described before, hydantoinase activity assays were carried out at 40 °C and 800 rpm, while assays employing d-Carb were conducted at 30 °C and 800 rpm. Samples were taken at selected reaction times, by placing the micro test tubes into the magnetic device for collecting the magnetic beads and withdrawing 100 μL of the supernatant. Depending on the analytical method, the samples were stopped by heat or by adding conc. HCl as described before. After sampling, the reaction vessel was gently mixed to get a homogeneous suspension and the reaction was continued. When the reaction was finished, bound proteins were eluted by withdrawing the supernatant, adding 500 μL elution buffer and incubating the suspension for 5 min at 800 rpm and 25 °C. The resulting eluate was transferred to a new micro test tube for determination of the protein concentration and for analysis by SDS-PAGE.

One unit (U) of hydantoinase activity is defined as the amount of enzyme that converts 1 µmol substrate per minute in catalysis buffer at 40 °C. However, the same applies to carbamoylase activity, determined at 30 °C. Due to the comparatively low activities of the hydantoinase as well as the low thermal and oxidative stability of the carbamoylase, activity assays were conducted at the particular optimum temperature of the enzymes. The different activity assays employing crude cell extract, purified enzymes and immobilized enzymes were performed at the same temperature for the respective enzyme.

### Analytical procedures

#### SDS-PAGE analysis

For sample preparation, the insoluble part after chemical cell disruption and centrifugation was resuspended in _dd_H_2_O by using twice the amount of the culture volume at the moment of harvesting. Every soluble part that had to be analyzed by SDS-PAGE was diluted to a protein concentration of 1 mg/mL with _dd_H_2_O and samples with lower protein concentrations were used without dilution. After disrupting the samples of the BioLector^®^ cultivations by sonication and subsequent centrifugation, the soluble parts were also diluted to a concentration of 1 mg/mL with _dd_H_2_O. The resulting insoluble pellets were resuspended in 300 μL _dd_H_2_O.

*Photometric assay* using Ehrlich’s reagent was carried out for detection of the serine derivative *N*-carbamoylserine. If the reaction had not been stopped by conc. HCl, the samples were mixed with conc. HCl in a ratio of 1:1 afterwards. After centrifugation for 5 min at 13,000 rpm, the supernatant was used for analysis. *N*-carbamoylserine was used for calibration. In triplicates, 50 μL of every sample and standard solutions were given into a microtiter plate and subsequently 30 μL of Ehrlich’s reagent were added. After mixing for 1 min at 800 rpm, the absorption at 430 nm was measured in a photometer to quantify the *N*-carbamoylamino acid.

*HPLC analysis* of PheHyd, *N*CPheGly and PheGly as well as of BnH, *N*CPheAla and PheAla was conducted on an Agilent 1200 system using a HyperClone ODS-C18 column (5 μm, 120 Å, 50 × 4.6 mm, Phenomenex). An isocratic flow method with 0.8 mL/min at 22 °C was used with a mobile phase consisting of 80% of a 0.1% H_3_PO_4_ solution and 20% methanol, modified after Werner et al. (Werner et al. 2004). 5 μL of the samples were injected without dilution and measurement was carried out at a wavelength of 210 nm.

### Accession numbers for nucleic acid sequences

d-hydantoinase gene from *A. crystallopoietes* DSM20117(Werner et al. 2004): BD287386.

d-Hyd(co): KY684077.

## Results

### Expression under oxygen deficiency

**Fig. 2:**
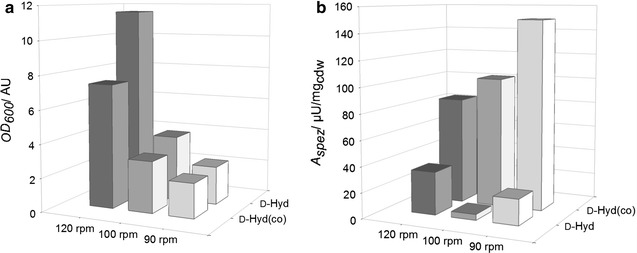
Cultivation of *E. coli* BL21DE3 and expression of d-Hyd and d-Hyd(co) under oxygen deficiency. **a** OD_600_ values of the d-Hyd and d-Hyd(co) cultures at the moment of harvesting after cultivation at 120 rpm (*dark grey*), 100 rpm (*grey*) and 90 rpm (*white*). **b** Specific activities of d-Hyd and d-Hyd(co) during whole cell biotransformations with 2 mM PheHyd as as substrate. The reactions were carried out at 40 °C and 800 rpm in Tris–HCl (50 mM, pH 8)

Experiments under oxygen deficiency have been carried out by using a higher culture volume (400 mL instead of 200 mL per 1 L baffled shaking flask) on the one hand and comparatively low shaking speeds (120, 100 and 90 rpm) on the other hand for the expression of d-Hyd and d-Hyd(co). For comparing enzyme activities after applying these different expression conditions, whole cell biotransformations were conducted. Figure 2a shows the different OD_600_ values of the cultures after 24 h, while Fig. 2b indicates their specific activities for the conversion of 2 mM PheHyd.

Regarding the OD_600_-values of the differently cultured *E. coli* BL21DE3 hosting the different recombinant hydantoinases, the values of the d-Hyd cultures are generally higher than that of the d-Hyd(co) cultures and the OD_600_ values decrease with decreasing rotation speeds for both cultures (Fig. 2a). At this juncture, the culture of the d-Hyd at 120 rpm exhibits a much higher OD_600_ of 11.54 compared to that of the d-Hyd(co) with an OD_600_ of 7.37. Compared to the cultures at 120 rpm, the OD_600_ values of the d-Hyd and the d-Hyd(co) cultures decrease to 3.89 and 3.06 for 100 rpm and the cultures at 90 rpm show OD_600_ values of 2.27 and 2.05. In contrast, the specific activities of the d-Hyd(co) cultures are much higher than that of the d-Hyd cultures (Fig. 2b). Here, the specific activity increases with decreasing rotation speed. The highest value for the d-Hyd(co) culture was determined at 90 rpm with 149.5 μU/mg_cdw_, while the lowest specific activity was 83.7 μU/mg_cdw_ at 120 rpm. As mentioned before, the specific activities for the d-Hyd are much lower with values from 4.50 to 33.4 μU/mg_cdw_ and with no noticeable tendency concerning the rotation speed.

**Fig. 3:**
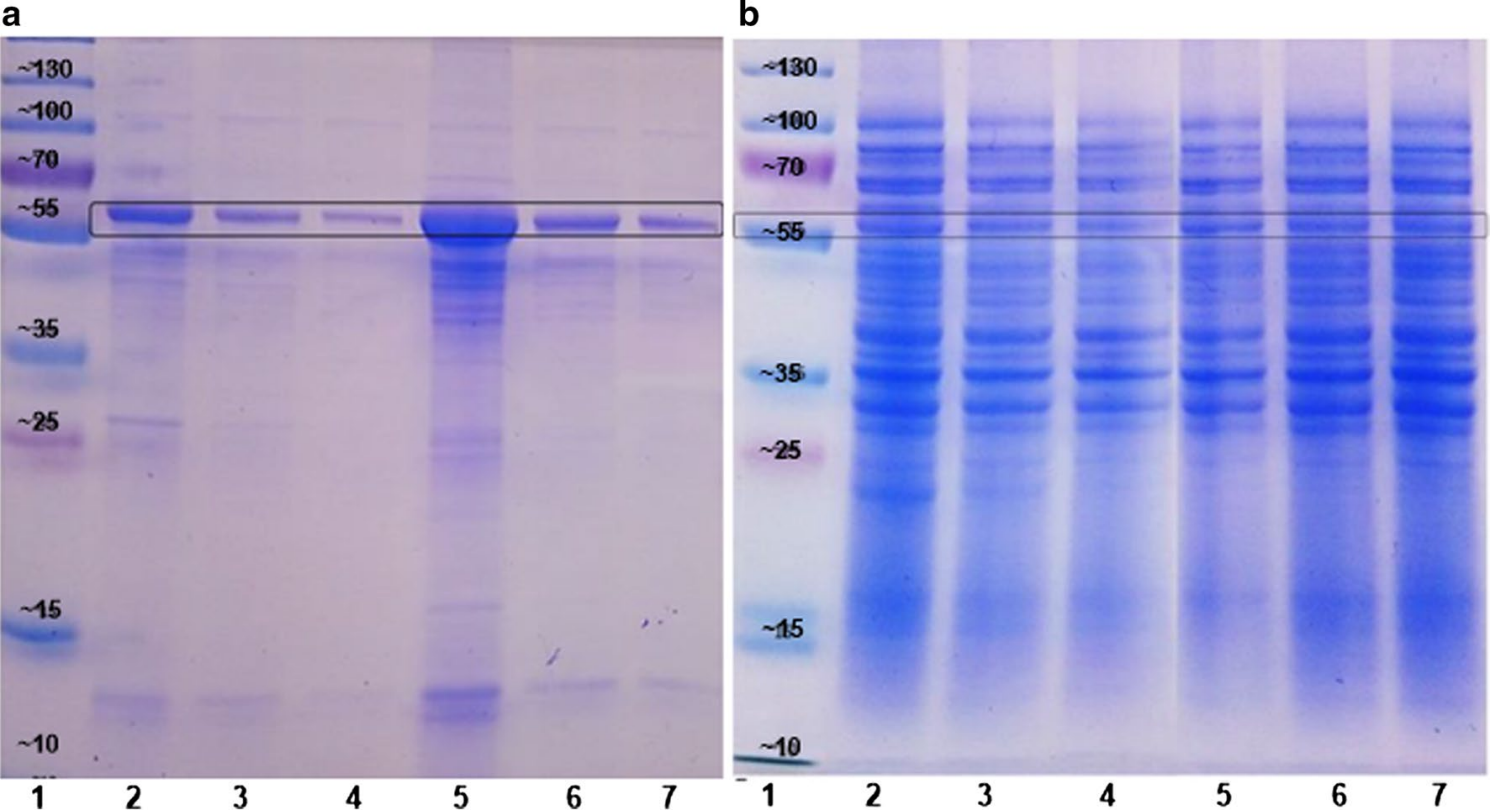
SDS-PAGE of *E. coli* BL21DE3 cultures hosting d-Hyd and d-Hyd(co) after cultivation and expression under oxygen deficiency. **a** Qualitative analysis of the insoluble fraction, *1* protein standard with molecular weights in kDa, *2* d-Hyd at 120 rpm, *3* d-Hyd at 100 rpm, *4* d-Hyd at 90 rpm, *5* d-Hyd(co) at 120 rpm, *6* d-Hyd(co) at 100 rpm, *7* d-Hyd(co) at 90 rpm. **b** Qualitative analysis of the soluble fraction, *1* protein standard with molecular weights in kDa, *2* d-Hyd at 120 rpm, *3* d-Hyd at 100 rpm, *4* d-Hyd at 90 rpm, *5* d-Hyd(co) at 120 rpm, *6* d-Hyd(co) at 100 rpm, *7* d-Hyd(co) at 90 rpm

To investigate the formation of inclusion bodies and the synthesis of the hydantoinase in its native and soluble state, qualitative analysis was conducted via SDS-PAGE (Fig. 3).

Insoluble fractions after cultivation under hypoxic conditions are shown in Fig. 3a. The molecular weight of the d-Hyd is around 52 kDa, while the d-Hyd(co) has a molecular weight of around 56 kDa. Both hydantoinase cultures show a clear band in every insoluble fraction with a suitable molecular weight for the enzyme, while bands for the d-Hyd (lane 2–4) are generally slightly thinner than bands for the d-Hyd(co) (lane 5–7). Additionally, the intensities of bands for insoluble hydantoinase decrease together with rotation speeds for the cultured bacteria. Figure 3b, image of the corresponding samples from crude cell extracts, shows an identical pattern with stained proteins about the entire molecular weight range for both recombinant hydantoinase cultures with every rotation speed for the cultured bacteria. Additionally, corresponding bands for the hydantoinases are visible in every sample with molecular weights of 52 kDa respectively 56 kDa.

### Screening for optimization of expression conditions

**Fig. 4:**
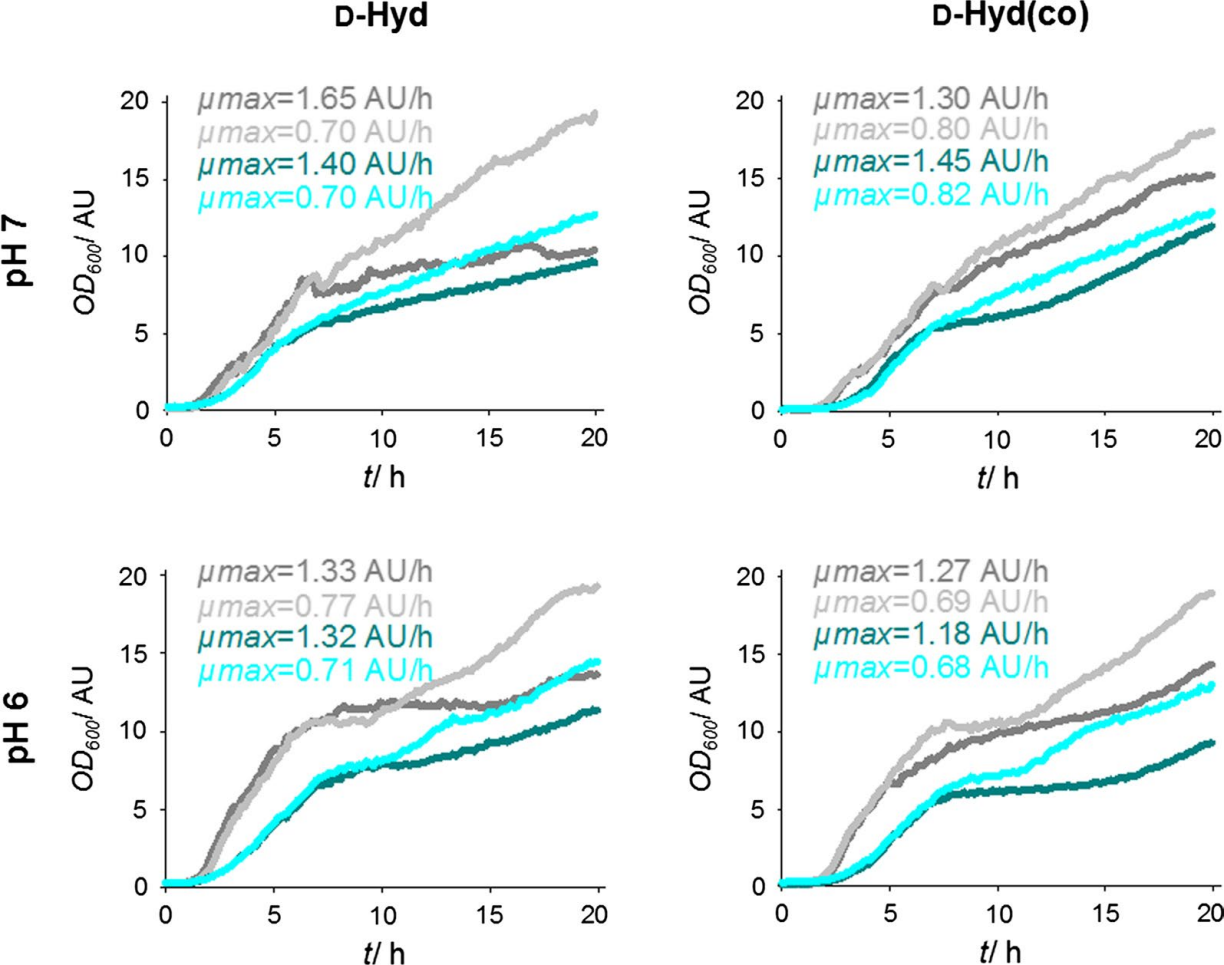
Screening for cultivation conditions of *E. coli* BL21DE3 expressing d-Hyd and d-Hyd(co). Growth curves and maximum growth rates under different cultivation conditions. -EtOH +IPTG (*dark grey*), −EtOH −IPTG (*grey*), +EtOH +IPTG (*dark cyan*), +EtOH −IPTG (*cyan*). Cultivations were carried out in triplicates, shown are the mean values

As a simple method to induce chaperones respectively heat shock proteins indirectly, cultivation and induction under physical stress was reported (Thomas and Baneyx 1996; Gasser et al. 2008). To allow the investigation of different factors, a screening was conducted using the BioLector^®^ microfermentation system (m2p-labs, Germany). Baumann et al. also investigated various cultivation conditions including the oxygen transfer rate by varying the shaking speed as well as the inducer concentration (Baumann et al. 2015). Since these experiments showed no influence on bacterial growth and product formation after variation of IPTG concentration, but very high product concentrations were observed without induction of the T7-promoter, in this work only induction with 1 mM IPTG as well as no induction were conducted. Concerning the shaking speed, 600 rpm resulted in highest protein expression for every tested setup, the rotation speed at which every experiment in this work was carried out. Due to the high throughput of different setups, assays were carried out as whole cell biotransformations and samples were taken after 0 and 24 h. Figure 4 shows the OD_600_ values during cultivation as well as the maximum growth rates of the cultures for both enzymes at every tested setup.

Regarding the OD_600_ values of the different cultures, it is noticeable that the non-induced cultures show higher growth rates in every case. The same applies to the cultures without EtOH compared to that with 3% EtOH. When comparing every cultivation setup for both hydantoinases, the d-Hyd(co) always shows higher OD_600_ values at the end of cultivation than the d-Hyd, but for the cultivation at pH 6 this observation is not distinct. Additionally, for the d-Hyd(co) the cultures at pH 6 have lower OD_600_ values at the end of culturing, while for the d-Hyd it is contrary. The tendencies are different for the maximum growth rates, since there is no distinction between induced and non-induced cultures. In contrast, cultures with and without 3% EtOH show major differences in maximum growth rates with a decrease of up to 50% for the cultures containing EtOH. For the d-Hyd(co), a slight decrease in the maximum growth rate is observed when culturing at pH 6 instead of pH 7, while for the d-Hyd no tendency is noticeable. In general, also no tendency is perceivable concerning the maximum growth rate between the strains expressing the two different plasmids.

**Fig. 5:**
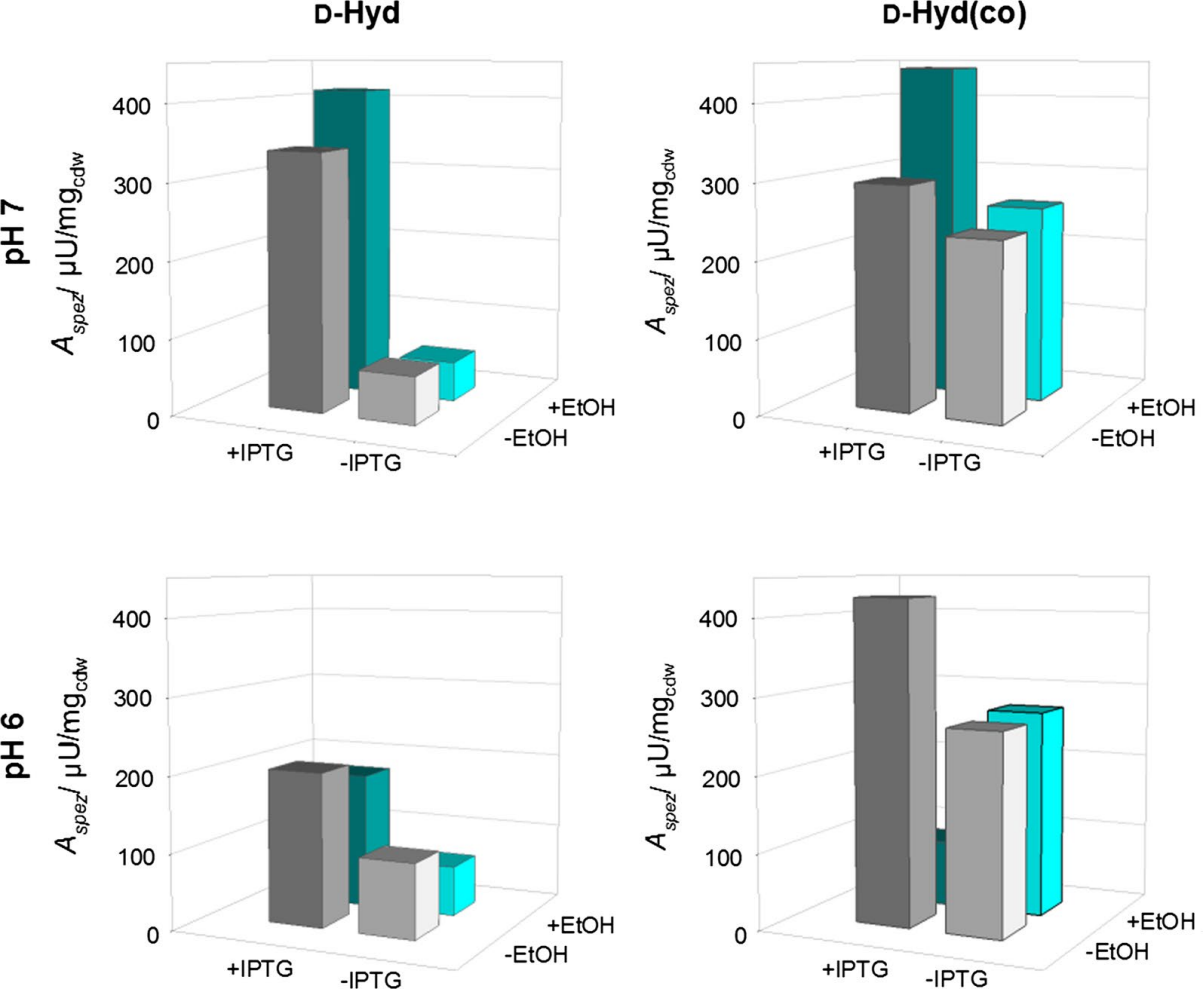
Screening for cultivation conditions of *E. coli* BL21DE3 expressing d-Hyd and d-Hyd(co). Specific activities after whole cell biotransformation with 2 mM PheHyd after cultivation and induction under different conditions. −EtOH +IPTG (*dark grey*), −EtOH −IPTG (*grey*), +EtOH +IPTG (*dark cyan*), +EtOH −IPTG (*cyan*)

To enable a statement, whether the recombinantly expressed hydantoinases are in their active form, whole cell biotransformations were carried out using PheHyd as a substrate. The specific activities for d-Hyd as well as d-Hyd(co) after cultivation and induction at different conditions are illustrated in 3D bar plots (see Fig. 5).

The determined specific activities for the d-Hyd(co) are higher than that of the d-Hyd for every cultivation setup. d-Hyd(co) exhibits the highest specific activity with 440.7 μU/mg_cdw_ at pH 7, induction with 1 mM IPTG and 3% EtOH, while the d-Hyd reaches with 411.1 μU/mg_cdw_ its highest specific activity at the same cultivation setup. In contrast, the lowest specific activity for the d-Hyd(co) is determined at pH 6, induction with 1 mM IPTG and 3% EtOH with 86.7 μU/mg_cdw_ and for the d-Hyd at pH 7, no induction and 3% EtOH with 51.6 μU/mg_cdw_. d-Hyd cultures that have not been induced show a significant decrease in specific activity for every culture condition compared to induced cultures, while the specific activities for non-induced d-Hyd(co) cultures show only slightly lower values compared to the induced ones. This result is not obtained for the d-Hyd(co) at pH 6 with 3% EtOH, in which the non-induced cultures show an increased specific activity compared to the induced ones. d-Hyd cultures containing EtOH exhibit decreased specific activities except for the d-Hyd at pH 7 with 1 mM IPTG. This is in contrast to the determined specific activities of the d-Hyd(co), which result in higher values when adding 3% EtOH to the culture media, except for the cultivation setup at pH 6 with induction. Regarding cultivations at different pH values, induced d-Hyd cultures show higher specific activities at pH 6, while non-induced cultures show higher specific activities at pH 7. For the d-Hyd(co), cultivation at pH 6 mostly results in higher specific activities than at pH 7, except for the cultivation at pH 7 with induction and 3% EtOH that results in the highest specific activity of all investigated setups.

**Fig. 6:**
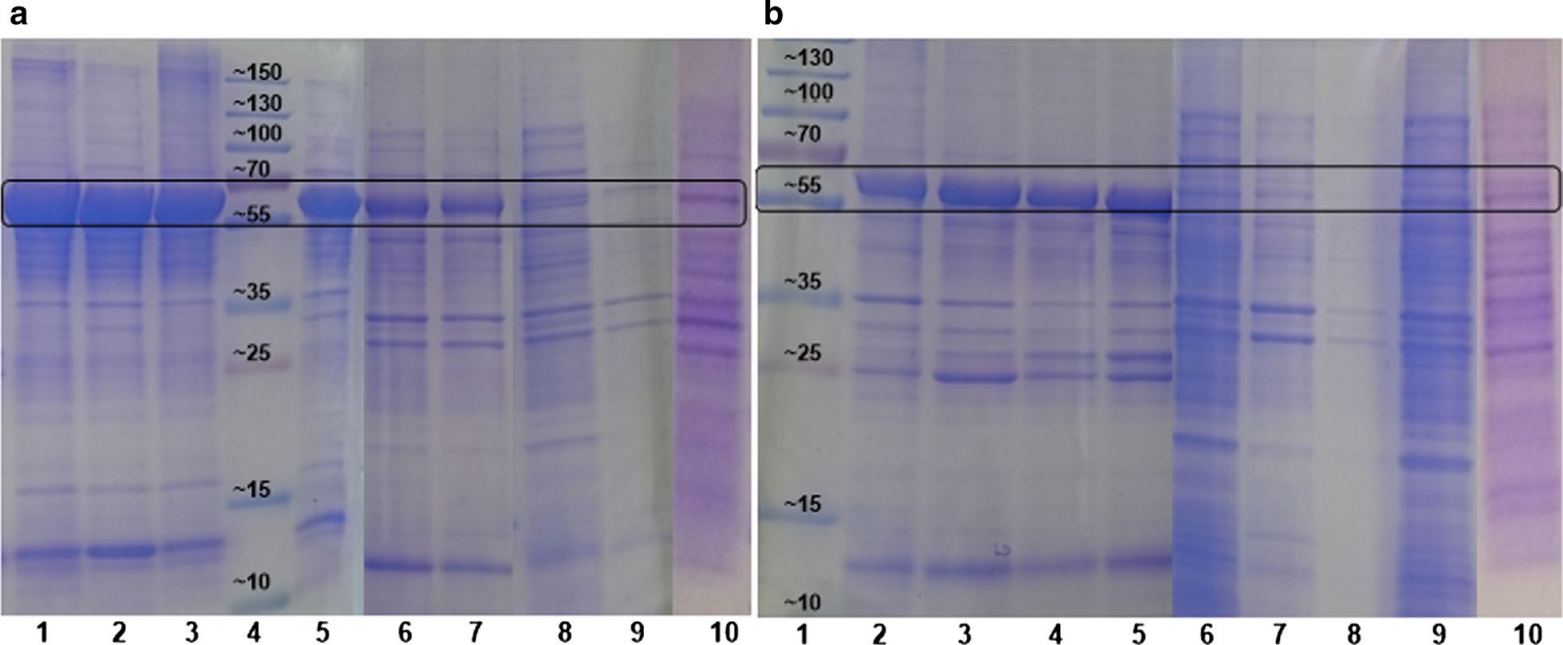
SDS-PAGE of cultures of *E. coli* BL21DE3 hosting d-Hyd(co) and d-Hyd after screening for cultivation conditions. **a** d-Hyd(co) insoluble fraction, *1* +IPTG −EtOH pH 7, *2* +IPTG −EtOH pH 6, *3* +IPTG +EtOH pH 7, *4* protein standard with molecular weights in kDa, *5* +IPTG +EtOH pH 6, *6* −IPTG −EtOH pH 7, *7* −IPTG −EtOH pH 6, *8* −IPTG +EtOH pH 7, *9* −IPTG +EtOH pH 6, *10* soluble fraction. **b** d-Hyd insoluble fraction, *1* protein standard with molecular weights in kDa, *2* +IPTG −EtOH pH 7, *3* +IPTG −EtOH pH 6, *4* +IPTG +EtOH pH 7, *5* +IPTG +EtOH pH 6, *6* −IPTG −EtOH pH 7, *7* −IPTG −EtOH pH 6, *8* −IPTG +EtOH pH 7, *9* −IPTG +EtOH pH 6, *10* soluble fraction

For a qualitative analysis of the investigated hydantoinases after cultivation and induction at various conditions, SDS-PAGE was conducted for every soluble and insoluble fraction after cell disruption and centrifugation (Fig. 6).

In Fig. 6a, insoluble fractions of the d-Hyd(co) are shown (lane 1–9), while Fig. 6b shows insoluble fractions of the d-Hyd (lane 1–9). Since the soluble fractions from different cultures resulted in the same band pattern and intensity for both enzymes, lane 10 shows exemplary the soluble fraction of the cultivation at pH 7, 1 mM IPTG and 3% EtOH in each case. Comparing the insoluble fractions of the d-Hyd and d-Hyd(co) cultures, the latter exhibit generally bands with higher intensity at 56 kDa for every investigated cultivation and induction setup. For the induced d-Hyd(co) cultures, the intensity of this band decreases with decreasing pH. The same cultures containing 3% EtOH have also smaller bands for the d-Hyd(co) than the cultures without EtOH. In contrast, the induced d-Hyd cultures show no difference in intensity for the bands at 52 kDa in the insoluble fractions. The non-induced d-Hyd(co) cultures show much less intensive bands than the induced ones. By comparing the soluble fractions of the d-Hyd(co) (Fig. 6a, lane 10) with those of the d-Hyd cultures (Fig. 6b, lane 10), the latter show a less intensive band for the hydantoinase in every case. The intensity of this band is generally very low for both enzymes and every cultivation setup.

### Purification of both enzymes via Ni Sepharose beads

Purification of d-Hyd(co) and d-Carb was conducted applying the above mentioned modified purification protocol for Ni Sepharose beads as well as buffer exchange using Slyde-A-Lyzer dialysis devices. Biotransformation assays with 5 mM PheHyd respectively 5 mM *N*CPheAla as a substrate were conducted to determine enzyme activities as well as recovery and purification factor. The corresponding results are listed in Table 1.

**Table 1:** Purification of the d-Hyd(co) and d-Carb via Ni Sepharose beads

Enzyme	Fraction	A (mU/mL)	A_vol_ (mU/mL)	A_tot_ (mU)	c_prot, tot_ (mg)	A_spez_ (mU/mg)	Recovery (%)	Purification fold
d-Hyd(co)	Crude extract	12.8	25.7	102.9	95.9	1.1	–	–
	Eluate 1^a^	1.2	2.4	4.9	0.9	5.2	4.7	4.9
	Eluate 2^a^	1.7	3.3	6.6	0.3	20.1	6.5	18.8
d-Carb	Crude extract	128.9	257.8	1031.0	61.4	16.8	–	–
	Eluate 1^a^	113.8	227.6	455.3	0.8	554.9	44.2	33.0
	Eluate 2^a^	59.9	119.7	239.4	0.8	300.0	23.3	17.9

^a^Eluate 1 and 2: subsequent elutions of the same pulldown

According to the biotransformation assay, the specific activity of the purified d-Hyd(co) in eluate 1 is with 5.2 mU/mg five times higher than the specific activity of the crude cell extract. Eluate 2 even shows an increase in activity to 20.1 mU/mg. For eluate 1, a recovery of 4.7% and a 4.9 fold purification are achieved, eluate 2 results in even higher values. For the d-Carb, the crude cell extract shows the lowest specific activity of 16.8 mU/mg, while eluate 1 has the highest specific activity of 554.9 mU/mg. Regarding recovery and purification factor, with 44.2% and 33.0, the first elution fraction also exhibits higher values than elution 2.

### Purification of both enzymes via functionalized magnetic beads

To obtain a quantitative statement about the success of the purification of d-Hyd(co) and d-Carb via magnetic beads, activity assays were conducted employing the eluate and either 5 mM PheHyd or 5 mM *N*CPheAla as a substrate (see Table 2).

**Table 2:** Purification of the d-Hyd(co) and d-Carb via functionalized magnetic beads

Enzyme	Fraction	A (mU/mL)	A_vol_ (mU/mL)	A_tot_ (mU)	c_prot, tot_ (mg)	A_spez_ (mU/mg)	Recovery (%)	Purification fold
d-Hyd(co)	Crude extract	7.7	15.5	15.5	11.97	1.3	–	–
	Eluate	1.8	3.7	1.8	0.04	46.4	11.9	35.9
d-Carb	Crude extract	76.4	152.9	152.9	15.57	10.0	–	–
	Eluate	49.7	99.4	49.7	0.04	1243.0	32.5	126.6

The specific activity of the d-Hyd(co) crude extract is 1.3 mU/mg, while for the eluate respectively purified d-Hyd(co), a specific activity of 46.4 mU/mg is determined and a recovery and purification factor of 11.9% and 35.9 were obtained. Results for the d-Carb revealed, that the crude cell extract has a specific activity of 10.0 mU/mg, while the purified d-Carb has a much higher specific activity of 1243.0 mU/mg, a recovery of 32.5% and a 126.6 fold purification.

**Fig. 7:**
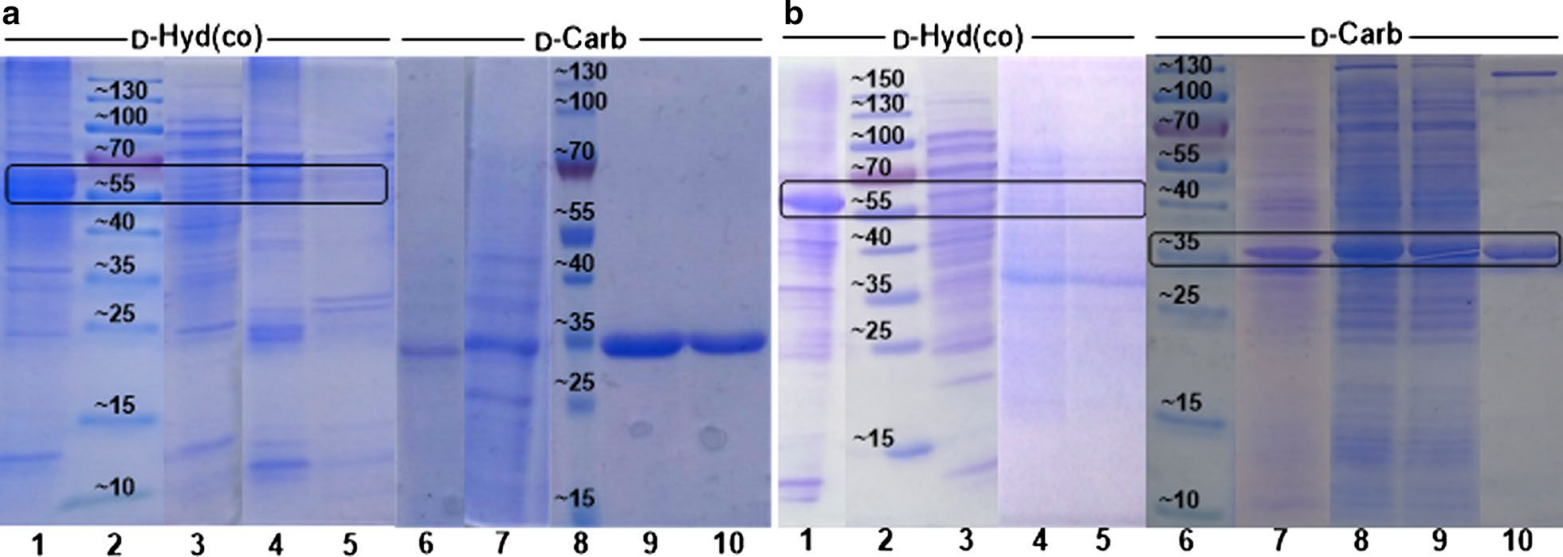
SDS-PAGE analysis of different fractions after purification of both enzymes. **a** Ni Sepharose beads *1*–*5* hydantoinase, *1* insoluble fraction, *2* protein standard with molecular weights in kDa, *3* crude extract, *4* eluate 1, *5* eluate 2, *6* **-** *10* carbamoylase, *6* insoluble fraction, *7* crude extract, *8* protein standard with molecular weights in kDa, *9* eluate 1, *10* eluate 2. **b** Functionalized magnetic beads, *1*–*5* hydantoinase, *1* insoluble fraction, *2* protein standard with molecular weights in kDa, *3* crude cell extract, *4* wash 1, *5* eluate *6*–*10* carbamoylase, *6* protein standard with molecular weights in kDa, *7* insoluble fraction, *8* crude cell extract, *9* wash 1, *10* eluate

Figure 7 shows the SDS-PAGE for the purification via Ni Sepharose beads as well as for the purification via functionalized magnetic beads.

SDS-PAGE analysis after purification of the d-Hyd(co) via Ni Sepharose beads (Fig. 7a) reveals a distinct band for the hydantoinase in eluate 1 (lane 4), while eluate 2 (lane 5) has generally lower intensities. The same method was applied for the d-Carb, at which both eluate 1 (lane 9) and eluate 2 (lane 10) show a very intense band for the carbamoylase. Compared to the results of the hydantoinase, no further bands were detected. Similar results were obtained for the purification of both enzymes via functionalized magnetic beads (Fig. 7b).

### Immobilization of both enzymes via functionalized magnetic beads

Immobilization of d-Hyd(co) was carried out and next to examination of the resulting specific activities toward PheHyd, two more substrates were investigated: Benzylhydantoin (BnH) as well as hydroxymethylhydantoin (HMH). For the synthesis of non-canonical amino acids via hydantoinase process in cell-free reaction systems using immobilized enzymes on magnetic beads, the d-Carb was also immobilized as described for the hydantoinase, whereat *N*-carbamoylphenylglycine (*N*CPheGly), *N*-carbamoylphenylalanine (*N*CPheAla) and *N*-carbamoylserine (*N*CSer) have been applied as substrates (see Table 3).

**Table 3:** Immobilization of d-Hyd(co) and d-Carb via functionalized magnetic beads and hydrolysis of different substrates

Fraction	Substrate	d-Hyd(co)		Substrate	d-Carb	
		c_prot_ (mg/mL)	A_spez_ (mU/mg)		c_prot_ (mg/mL)	A_spez_ (U/mg)
Crude extract	PheHyd	5.00	0.36	*N*CPheGly	5.00	0.07
Immobilized enzyme		0.11	70.75		0.03	11.75
Crude extract	BnH	5.00	0.28	*N*CPheAla	5.00	0.10
Immobilized enzyme		0.08	63.20		0.03	12.91
Crude extract	HMH	5.00	0.76	*N*CSer	5.00	0.08
Immobilized enzyme		0.08	105.47		0.03	11.75

The activity assay resulted in the highest specific activity for the immobilized d-Hyd(co) converting HMH with 105.47 mU/mg, while the lowest specific activity was determined for the conversion of BnH with 63.20 mU/mg. The same tendency is observed for specific activities of the crude cell extracts, which exhibit up to 200 times lower specific activities compared to the immobilized d-Hyd(co).

Biotransformation assays with different substrates reveal a successful immobilization of the d-Carb with similar specific activities, whereat the substrate *N*CPheAla was converted with the highest specific activity of 12.91 U/mg. The specific activity of the crude cell extracts range from 0.07 U/mg for the conversion of *N*CPheGly to 0.10 U/mg for the conversion of *N*CPheAla.

## Discussion

### Expression under oxygen deficiency

Since enhanced solubility of recombinant proteins was reported at low temperatures and weak doses of inductor (Carrió and Villaverde 2002; Martínez-Alonso et al. 2009), both parameters were investigated in this work, but no improvement in soluble hydantoinase expression was observed (data not shown). This is consistent with the findings of Baumann et al., who showed that variation of the inductor concentration had no distinctive influence on bacterial growth and protein expression of the investigated target molecule. The same applied to the cultivation temperature, while the shaking speed was found to be more influencing concerning these matters (Baumann et al. 2015). On the basis of these investigations, experiments under oxygen deficiency have been carried out.

The fact that lower rotation speeds and consequently reduced oxygen transfer rates lead to decreased growth of the cultures is already common, since *E. coli* grows preferably by aerobic respiration. Nevertheless, at lower rotation speeds like 100 and 90 rpm, the difference in this work is not that crucial, since the growth is already very slow (Fig. 2a). More noticeable is the fact that the growth of the d-Hyd(co) culture was much slower than that of the d-Hyd culture at 120 rpm. This implies a stronger expression of the recombinant hydantoinase after codon-optimization, given that the growth is reduced and due to high expression levels. The determined specific activities of the d-Hyd(co) are in each case much higher than those of the d-Hyd, which confirms this assumption. It was observed that the specific enzyme activity of the d-Hyd(co) increased with decreasing rotation speed, which certainly results from slower growth rates as well as expression which is accompanied by correct folding of the enzyme (Strandberg and Enfors 1991; Baumann et al. 2015). Georgiou et al. reported that the time-scale for the folding of proteins may vary from milliseconds to days, depending on the amount of kinetic barriers that are included in the folding process (Georgiou and Valax 1996). The latter are, for example, caused by covalent reactions like the formation of disulfide bonds or *cis/trans* isomerizations and result in the accumulation of folded intermediates containing exposed hydrophobic surfaces that promote self-assembly. Since this self-assembly leads to the formation of inclusion bodies, it was supposed that decelerated expression of the target molecule leads to more correct folded proteins and higher specific activities (Bowden et al. 1991; Wetzel 1994). Probably due to the differences in codon-usage or caused by the high proliferation as well as expression rates, the specific activities of the not codon-optimized d-Hyd were comparatively low for all cultivation conditions. Consequently, there was no tendency perceivable regarding the different rotation speeds during cultivation and expression.

The results from SDS-PAGE, implying that the d-Hyd(co) shows high expression rates that mostly result in inclusion bodies, stay in contrast to the determined specific activities of this enzyme (see Fig. 3b). Since the latter were found to be very high compared to the d-Hyd, it was shown that despite the major amount of inclusion bodies, a clear improvement was achieved. Therefore, it was assumed that prevention of inclusion body formation should even lead to a further improvement. As demonstrated by the above discussed experiments, the codon-optimization resulted in a much higher expression of recombinant hydantoinase. These non-physiological amounts of produced proteins promote the intermolecular association of exposed hydrophobic surfaces before completion of the protein folding, which causes precipitation of folding intermediates (King et al. 1996; Carrió and Villaverde 2002). Since the direct or indirect induction of chaperones was reported to improve the expression of target molecules in their native state (Georgiou and Valax 1996; Thomas and Baneyx 1996), a screening for different expression conditions concerning these topics was conducted (see Additional file 1).

### Screening for optimization of expression conditions

The observance that non-induced cultures had higher OD_600_ values at the end of culturing in every case suggests that the proliferation is decreased after induction, since the resources are used for gene expression instead of growth. This is a fact, although the induction was conducted in a later growth phase (OD_600_ = 4) according to Baumann et al., since earlier induction would have led to faster protein expression and therefore also fast and poor folding of the target molecule (Georgiou et al. 1994; Georgiou and Valax 1996; Baumann et al. 2015). The d-Hyd of non-induced cultures showed a significant decrease in specific activities compared to that of induced cultures, while for the d-Hyd(co) of non-induced cultures only a slight decrease in specific activity was observed. Due to the optimized codon bias of the d-Hyd(co) for *E. coli*, basal activity of the T7-promoter in non-induced cultures was already sufficient to produce a high amount of native target molecule.

The addition of EtOH had a great influence on the final OD_600_ values as well as the maximum growth rates, since the resulting physiological stress led to a decrease in proliferation. The tendency of decreasing specific activities for the d-Hyd when cultured with EtOH is due to the general low expression level. This observation was contrary for the d-Hyd(co) cultured with 3% EtOH in the cultivation media, which showed higher specific activities than the d-Hyd(co) that had been cultured without EtOH. According to Thomas et al., the exposure to physiological stress like the addition of EtOH indirectly induces the expression of chaperones that assist in the correct folding of the target molecule (Thomas and Baneyx 1996). Only the cultivation setup at pH 6 with induction shows a decrease in specific activity, which is propably resulting from the chemical and metabolic stress at these conditions, leading to a decrease in expression of soluble d-Hyd(co).

The cultivation and induction at different pH values was investigated on the one hand to cause physiological stress for indirect induction of chaperones and on the other hand to possibly prevent degradation of the target protein by proteases at a lower pH value (Thomas and Baneyx 1996; Gasser et al. 2008). The fact that the specific activity of the d-Hyd cultured at the lower pH of 6 only increased for the induced cultures, while for the non-induced cultures a lower pH resulted in a decrease in specific activity of the d-Hyd, is also resulting from the generally low expression rates. This theory was supported by the determined specific activities of the d-Hyd(co), which by trend show higher specific activities at pH 6. The exception represents the cultivation setup at pH 7 with induction and 3% EtOH with the highest specific activity of all investigated setups. This suggests that combining both parameters that cause chemical stress affected the proliferation as well as protein expression so much that potentially improved folding of the target molecule carried no weight. When comparing maximum growth rates and specific activities, it was shown that the theory of a more correct protein folding at lower maximum growth rates and therefore lower expression rates (Baumann et al. 2015), was only consistent for the codon-optimized hydantoinase d-Hyd(co), which generally exhibits higher expression levels.

Concerning SDS-PAGE analysis, protein concentrations of the insoluble fractions were not definable by photometric assays and therefore, the pellets were diluted according to the culture volume. Consequently, since the final OD_600_ values of the induced d-Hyd(co) cultures decrease from line 1 to line 5 from 15.18 to 9.26 (Fig. 4a), the reduced intensity of the bands at 56 kDa for the hydantoinase are caused by lower OD_600_ values at the time of harvesting. The same applies to the non-induced cultures expressing d-Hyd(co) (Fig. 4a, lane 6–9), since cultures containing EtOH resulted in lower final OD_600_ values and also show bands with lower intensity than cultures without EtOH. Nevertheless, compared to the induced cultures expressing d-Hyd(co), much less inclusion bodies were detected although their final OD_600_ values were higher. This indicates that the basal activity of the T7-promoter was sufficient for the expression of d-Hyd(co) and additionally, the decreased expression rates resulted in improved folding of the target molecule (Gasser et al. 2008). However, when comparing the specific activities of induced and non-induced cultures, the non-induced cultures mostly exhibit lower values. This points out, that the lower concentration of inclusion bodies simply results from lower expression rates of the d-Hyd(co) instead of improved protein folding.

The same conclusions apply to the SDS-PAGE of induced and non-induced d-Hyd cultures. Additionally, it was observed that the intensity of the hydantoinase bands for every insoluble fraction of the d-Hyd culture are less intensive than hydantoinase bands of the d-Hyd(co) cultures, which can result on the one hand from lower final OD_600_ values and on the other hand from lower expression levels of the not codon-optimized enzyme. Referring to the soluble fractions of every cultivation setup, for both enzymes no differences in the pattern or intensity of the bands were noticeable, which is why for each enzyme only one setup is shown (Fig. 6a, b, lane 10).

Nevertheless, two important conclusions can be drawn due to SDS-PAGE analysis: On the one hand, a more intensive hydantoinase band for d-Hyd(co) compared to the d-Hyd suggests an increased amount of native hydantoinase for the codon-optimized version, which verifies the above discussed results. On the other hand, it is noticeable that the major part of expressed hydantoinase occurs as inclusion bodies for both enzymes and every tested cultivation setup.

Consequently, although the specific activity of both enzymes was improved successfully by indirect induction of chaperones or decelerated proliferation and expression rates, a further improvement of soluble expression was tested by coexpression of five different chaperone sets (see Additional file 1). Activity assays revealed the same tendency concerning the best cultivation setup, which was the cultivation at pH 7 with induction and with 3% EtOH in every case (see Additional file 1: Figure S1). Though, even the chaperone set C2 with the best results concerning specific activities is only half as active as the d-Hyd(co) without coexpression of chaperones with same cultivation conditions. Despite the fact that the coexpression of chaperones had been applied successfully in many cases and even for hydantoinases (Cai et al. 2009), it has not been effective in every case. Many possible reasons have been discussed, like enhanced proteolytic activities upon overexpression of chaperones (Straus et al. 1990; Kandror et al. 1994, 1999; Kondo and Nishihara 2000; Nishihara et al. 2000; García-Fruitós et al. 2007; Zahrl et al. 2007). These findings may explain the fact that in this case, no insoluble d-Hyd(co) as well as almost no soluble d-Hyd(co) was detectable (see Additional file 1: Figure S2).

Additionally, purification of occuring inclusion bodies was tested after the protocol of Diener et al. (2015), but no activity was detectable.

### Scale-up

For scaling up the culture volume, the cultivation setup at pH 7, induction with 1 mM IPTG and with addition of 3% EtOH was used for the d-Hyd(co). Since this cultivation setup was investigated for a 1 mL culture in a 48-well flowerplate at 600 rpm shaking frequency and 37 °C, it needed to be scaled up by keeping the oxygen transfer rate constant. Baumann et al. reported that 1 mL filling volume at 600 rpm in a flower plate corresponds to a kLa value of 88.9/h. For a 2.5 L baffled Tunair shake flask with 800 mL filling volume, they determined an incubator shaking speed of 180 rpm for equivalent oxygen uptake (Baumann et al. 2015). Based on these results, 1 L shaking flasks were filled up to 200 mL and shaken at 120 rpm to achieve a similar oxygen transfer rate.

Whole cell biotransformation with 2 mM PheHyd as a substrate resulted in a specific activity of 304.0 μU/mg_cdw_. The values for 1 mL culture volume in a flower plate at 600 rpm were an OD_600_ of 11.94 and a specific activity of 440.7 μU/mg_cdw_. Considering the altering conditions comparing a microcultivation of 1 mL with an upscale to 200 mL culture volume, the slight decrease of final OD_600_ as well as specific activity is acceptable. Based on these experiments, this cultivation setup was used for every further investigation concerning the purification as well as immobilization of the codon-optimized hydantoinase from *A. crystallopoietes* DSM 20117.

### Purification of both enzymes via Ni Sepharose beads

The results confirmed a successful purification of the d-Hyd(co). Although SDS-PAGE analysis of eluate 2 showed a very slight band for the hydantoinase compared to the corresponding band in eluate 1 (see Fig. 7a), this fraction revealed a much higher specific activity which was not caused by the lower protein concentration, since the volumetric activity of eluate 2 is higher, too. Consequently, it was shown that two elution steps allow purification with insignificant amounts of contamination. Xu et al. (2003) investigated the purification of the hydantoinase from *Burkholderia pickettii* via His-tag with a specific activity of 1.13 U/mg toward d,l-hydroxyphenylhydantoin, a recovery of 16.6% and a 2.6 fold purification. Although the specific activity of the investigated hydantoinase is comparatively low in our work, the recovery as well as purification factor is likewise when looking at both obtained elution fractions. Siemann et al. and Werner et al. investigated the purification of the same hydantoinase as in this work and every purification method was reported to result in higher specific activities (Siemann et al. 1999; Werner et al. 2004). However, the obtained recoveries and purification factors are comparable to the results of this work. Since both groups used methods like hydrophobic interaction chromatography in contrast to affinity tags and the other values are similar, the lower specific activities are suggested to result from the inserted tags. Ragnitz et al. reported a loss in activity of 90% for the hydantoinase from *Arthrobacter aurescens* DSM3747 after purification with a TALON^®^ column by His-tag (Ragnitz et al. 2001b). One possibility for the comparatively low enzymatic activity of the purified d-Hyd(co) is the negative influence of the His-tag itself, extracting the essential zinc ions from the catalytic center of this metalloenzyme. Additionally, the fact that two tags are attached to the hydantoinase may influence activity of an enzyme, since the location of affinity tags (*C*- or *N*-terminus) is sometimes crucial (Skerra and Schmidt 1999). The purification via IMAC chemistry as well as effects of metal ions on hydantoinase purification was investigated by Ko et al. who also obtained higher specific activities for a hydantoinase, but the purification factors were much lower. The highest specific activity as well as recovery was obtained using zinc ions for immobilization, while the nickel ions resulted in the second best values (Ko et al. 2011).

Concluding achievements of this work, a purification protocol was optimized for the purification of the d-Hyd(co), allowing a simple upscale of this method due to batch purification. Additionally, a moderate enzyme activity and good recovery as well as purification factor was achieved.

It was conspicuous that the crude extract of the d-Carb exhibited a much higher specific activity than the crude extract of the d-Hyd(co) with 1.1 mU/mg (see Table 1). SDS-PAGE showed that the purified d-Carb exhibits less non-specifically bound proteins than the d-Hyd(co), which is caused by the higher concentration of soluble enzyme in the crude cell extract, since low concentrations of the target molecule promote non-specific binding (see Fig. 7a). Consequently, this is a possible explanation for the comparatively low specific activity of d-Hyd(co), although these enzymes catalyze two different reactions. Furthermore, eluate 1 of the d-Carb purification process revealed a 33 times higher specific activity than the crude extract. The values for specific activity, recovery and purification factor of eluate 2 were lower than for eluate 1, but still very high. Taking together both eluates, a very high recovery of 67.5% was achieved by purification of the d-Carb via His-tag. Purification of the l-carbamoylase from *A. aurescens* via Streamline DEAE and MonoQ resulted in much lower values for the recovery as well as for the purification factor (Pietzsch et al. 2000), while Chen et al. achieved generally higher values by purification of the carbamoylase from *Agrobacterium radiobacter* via His-tag based affinity chromatography (Chen et al. 2003). Lower specific activities of the carbamoylase may result from its oxidative sensitivity as well as thermal instability. In 1979, Olivieri et al. discovered the involvement of thiol groups in d-carbamoylase activity and Grifantini et al. confirmed these findings by stating oxidative sensitivity via sequence analysis and mutagenesis experiments (Olivieri et al. 1979; Grifantini et al. 1996). Oxidation of cysteine thiol groups leads to inactiviation of the catalytic center of carbamoylases, which can be avoided by addition of reducing agents like DTT or β-mercaptoethanol (Buson et al. 1996; Louwrier and Knowles 1996). In this work, 5 mM DTT were added to every buffer solution during purification as well as during activity assays.

### Purification of both enzymes via functionalized magnetic beads

The results revealed a successful purification of the d-Hyd(co) by functionalized magnetic beads with a higher specific activity of the isolated d-Hyd(co) than for the purification using Ni Sepharose beads. The recovery was nearly the same when comparing both eluates of the Ni Sepharose purification with the purification by magnetic beads, but the purification factor was higher with the latter method (see Table 2). Since the incubation times are much shorter using the protocol for purification via functionalized magnetic beads, it was suggested that the hydantoinase was maybe exposed to proteolytic digestion during the incubation of the crude cell extract with the Ni Sepharose beads in spite of the added protease inhibitor. Compared to the investigations of Ko et al. dealing with the purification of the hydantoinase via IMAC chemistry using different metal ions, the specific activities are relatively low, but the purification factor as well as recovery are much higher (Ko et al. 2011). The same applies to the work of Xu et al. (2003) whereat the purification factor was lower, but the resulting specific activities were higher than in the shown results. This verifies the suitability of this method for the purification of the d-Hyd(co) regarding the obtained purification factor as well as recovery. A possible explanation for the comparatively low specific activities maybe result from the *N*-terminal SBP-tag as already discussed before.

Concerning the d-Carb, the determined specific activities proved also a successful purification using functionalized magnetic beads with a high recovery as well as purification factor. The recovery was lower compared to the purification via Ni Sepharose beads (around 68%), while the purification factor was about 2.5 times higher for the purification of the d-Carb via functionalized magnetic beads. The lower recovery for this method can be explained by the large band for the d-Carb remaining in the supernatant after incubation with the beads (see Fig. 7b). The longer incubation times of the Ni Sepharose protocol may cause a loss in d-Carb activity due to oxidation as well as thermal instability (Oh et al. 2002; Chiang et al. 2008). These facts highlight the advantages of using functionalized magnetic beads for purification directly from the crude cell extract. Nevertheless, magnetic beads are comparatively expensive and high batches for the purification of enzymes would cause very high costs and is therefore not profitable for industrial applications. Pietzsch et al. purified the l-carbamoylase from *A. aurescens* using IMAC chromatography with a specific activity of 5.9 U/mg, a yield of 55% and a purification factor of 11.3 (Pietzsch et al. 2000). Compared to the results shown above, the achieved specific activity and yield were slightly higher, while the purification factor was much lower. The very high purification factor is suggested to result on the one hand from higher stability of the d-Carb in the immobilized form an on the other hand from very short incubation times when immobilizing the d-Carb directly from the crude cell extract. Furthermore, no previous purification of the enzyme is necessary, avoiding loss of enzyme and activity during purification steps.

### Immobilization of both enzymes via functionalized magnetic beads

Due to their thermal instability as well as oxidative and proteolytic sensitivity many efforts have been made for the immobilization of hydantoinases and carbamoylases with visible success (Pietzsch et al. 1998; Ragnitz et al. 2001b; Chiang et al. 2008; Nandanwar et al. 2013). Most investigations are dealing with encapsulation, covalent immobilization or non-covalent adsorption techniques. These methods exhibit not only advantages, but also drawbacks like enzyme leakage for encapsulation methods or a loss in enzyme activity upon covalent binding. Therefore, the immobilization via affinity tags displays a promising alternative. In this work, the immobilization via non-covalent adsorption by coordination bonds between immobilized metal ions and amino acids of the target molecule was investigated, which avoids mentioned disadvantages.

By conducting activity assays, it was shown that the direct immobilization of the d-Hyd(co) from the crude cell extract to functionalized magnetic beads via His-tag was successful (see Table 3). The specific activity of the enzyme was increased by up to 200% upon immobilization. Additionally, the obtained specific activities are much higher than of the purified d-Hyd(co) with a maximal specific activity of 20.1 mU/mg, which is probably caused by a higher stability of the d-Hyd(co) upon immobilization. Furthermore, the reported loss in activity due to attachment of a His-tag to this enzyme can be prevented by immobilization and therefore occupation of this tag, which could otherwise extract the zinc ions from its active center (Ragnitz et al. 2001b). As already discussed, the immobilization employing affinity tags displays an advantage compared to covalent immobilization methods or, for example, immobilization via polyglutaraldehyde particles entrapped in calcium alginate beads, since this method resulted in partial inactivation of the hydantoinase as well as induced mass transfer resistance and consequently lower reaction rates (Fan and Lee 2001).

Immobilization of the d-Carb resulted in very high specific activities compared to the crude cell extract with around 130 fold increased specific activities. Conversion of *N*CPheAla resulted in the highest specific activities, but the other subtrates were converted in almost the same manner. The fact that the eluted d-Carb fraction contains less impurities than the d-Hyd(co), suggests that the immobilization occurred more specifically for the target molecule due to the higher expression of soluble d-Carb (see Fig. 7b). Comparing this immobilized d-Carb with the activities of the enzyme purified by Ni Sepharose beads, the resulting specific activities were much higher, indicating an increased thermal as well as oxidative stability of the d-Carb upon immobilization as also reported from other groups (Oh et al. 2002; Chiang et al. 2008). Until now, immobilization of a carbamoylase via IMAC chemistry is not reported. However, immobilization of the l-carbamoylase from *Bacillus kaustophilus* on Eupergit C was investigated and a lower optimal specific activity of 2.91 U/mg was achieved (Yen et al. 2010). Immobilization of the l-carbamoylase from *Geobacillus stearothermophilus* CECT43 on Sepabeads EC-HFA/S was reported to result in a maximum specific activity of around 14.00 U/mg by Soriano-Maldonado et al. (2014) which is comparable to our results.

Recyclation of the immobilized d-Carb was also tested by resuspending the used magnetic beads carrying the d-Carb in 500 µL catalysis buffer and adding 500 µL of the substrate solution (see “Materials and methods”, Biotransformation assays). This resulted in no activity, suggesting a very fast oxidation and therefore inactivation of the d-Carb (Buson et al. 1996; Grifantini et al. 1996; Louwrier and Knowles 1996).

Since the use of DTT is not recommended for the functionalized magnetic beads, no reducing agent was applied for preventing oxidation of the active center and therefore inactivation of this enzyme occurred.

The investigated method for immobilization of the d-Carb is very simple, fast and results in better or comparable specific activities of the d-Carb although comparison with other reported results is difficult due to the varying applied methods. But this fact shows that there is much more potential in optimization of the immobilization of carbamoylases by coordination with metal ions. Furthermore this method is more gentle due to the abdication of covalent binding techniques that often cause loss in enzyme activity, plus transport limitations that occur using encapsulation methods hold off.

Since a more specific as well as stable affinity was reported for the application of SBP-tag compared to His-tag (Lee et al. 1996; Voss and Skerra 1997), the hydantoinase from *A. crystallopoietes* DSM20117 was additionally provided with an *N*-terminal Strep-tag. Especially regarding immobilization of the hydantoinase toward applications in microfluidic reaction systems, this affinity tag should be helpful.

However, every immobilization approach for the d-Hyd(co) on Dynabeads^®^ M-280 Streptavidin resulted in no determinable enzyme activity for the three tested substrates PheHyd, BnH as well as HMH (data not shown). Since the purification approaches in this work already showed a decreased enzyme activity for the isolated d-Hyd(co) compared to the immobilized enzyme, it was suggested that occupation of the His-tag by immobilization employing this tag results in shielding of these histidine residues from the catalytic center of the enzyme. Consequently, the histidines of the His-tag are not able to withdraw the zinc ions that are required for an active hydantoinase. Ragnitz et al. (2001b) also reported a loss in activity of up to 90% upon purification of a hydantoinase via His-tag. This would also explain the complete loss in enzyme activity upon immobilization of the hydantoinase at the *N*-terminus, which leads to an exposure of the *C*-terminal His-tag and therefore promotes withdrawal of the zinc ions from the active center.

To enable a better overview of the results regarding purification and immobilization, Table 4 shows the values determined in this work compared to values from literature.

**Table 4:** Overview of investigated purification and immobilization methods for both enzymes and comparison to other works

	Via	Enzyme	Recovery (%)	Purification fold	References
Ni Sepharose purification	*Ni Sepharose beads*	*Hyd*	*11.2*	*23.7*	*This study*
	IMAC chromatography	Hyd	16.6	22.6	Xu et al. (2003)
	*Ni Sepharose beads*	*Carb*	*67.5*	*50.9*	*This study*
	IMAC chromatography	Carb	70.0	–	Chen et al. (2003)
Functionalized magnetic bead purification	*Functionalized magnetic beads*	*Hyd*	*11.9*	*35.9*	*This study*
	IMAM^a^	Hyd	3.5	6.8	Ko et al. (2011)
	*Functionalized magnetic beads*	*Carb*	*32.5*	*126.6*	*This study*
	IMAC chromatography	Carb	55.0	11.3	Pietzsch et al. (2000)

Values for the hydantoinase and carbamoylase determined in this work (*italics*) compared to values reported in literature using other hydantoinases and carbamoylases (*regular*). Ni Sepharose purification: elution 1 + elution 2

^a^Immobilized metal ion affinity membrane

It has to be noted that every purification approach using metal ion affinity was compared with other works using IMAC chromatography for purification instead of batch approaches, since no such approaches have been reported yet. For immobilization using metal ion affitnity, the most comparable approach was the work from Ko et al. (2012) applying an immobilized metal ion affinity membrane for the immobilization of the hydantoinase. Regarding immobilization of the carbamoylase, there is nothing reported using metal ion affinity until now, therefore an approach employing Eupergit C was utilized for comparison (Yen et al. 2010).

By means of this work, a promising basis for the application of the hydantoinase process in cell-free reaction systems was accomplished. After improvement of the expression conditions, a hydantoinase and carbamoylase was successfully purified with two different methods. Furthermore, an immobilization method using functionalized magnetic beads was established, which enables the immobilization of both enzymes directly from the crude cell extract. The increasing specific activities upon immobilization as well as the conversion of different substrates by both enzymes point to the potential applicability of this system for the synthesis of optically pure α-amino acids.

## Additional file

**Additional file 1.** Additional figures.

## Abbreviations

BnH: benzylhydantoin
d-Carb: carbamoylase from *A. crystallopoietes* DSM 20117 with *C*-terminal His-tag
d-Hyd: hydantoinase from *A. crystallopoietes* DSM 20117 with *C*-terminal His-tag
d-Hyd(co): codon-optimized hydantoinase from *A. crystallopoietes* DSM 20117 with *C*-terminal His-tag and *N*-terminal SBP-tag
HMH: hydroxymethylhydantoin
*N*CPheAla: *N*-carbamoyl-α-phenylalanine
*N*CPheGly: *N*-carbamoyl-α-phenylglycine
*N*CSer: *N*-carbamoyl-α-serine
PheHyd: phenylhydantoin
