# Supplementary material for: Toward a cell-free hydantoinase process: screening for expression optimization and one-step purification as well as immobilization of hydantoinase and carbamoylase
Source: AMB Express. 2017 Jun 9;7:122. doi: 10.1186/s13568-017-0420-3 (PMC5466576; doi:10.1186/s13568-017-0420-3)
Supplement: Supplementary file 1 — Additional file 1. Additional figures. [file 13568_2017_420_MOESM1_ESM.pdf]

Supplementary Material

Toward a cell-free hydantoinase process: Screening for expression optimization and one-step purification as well as immobilization of hydantoinase and carbamoylase

Christin Slomka*, Georg Paris Späth, Phillip Lemke, Marc Skoupi, Christof M. Niemeyer, Christoph Syldatk, Jens Rudat

*christin.slomka@kit.edu, Karlsruhe Institute of Technology (KIT), Institute of Process Engineering in Life Sciences, Section II: Technical Biology, Engler-Bunte-Ring 3, 76131 Karlsruhe, Germany

AMB Express

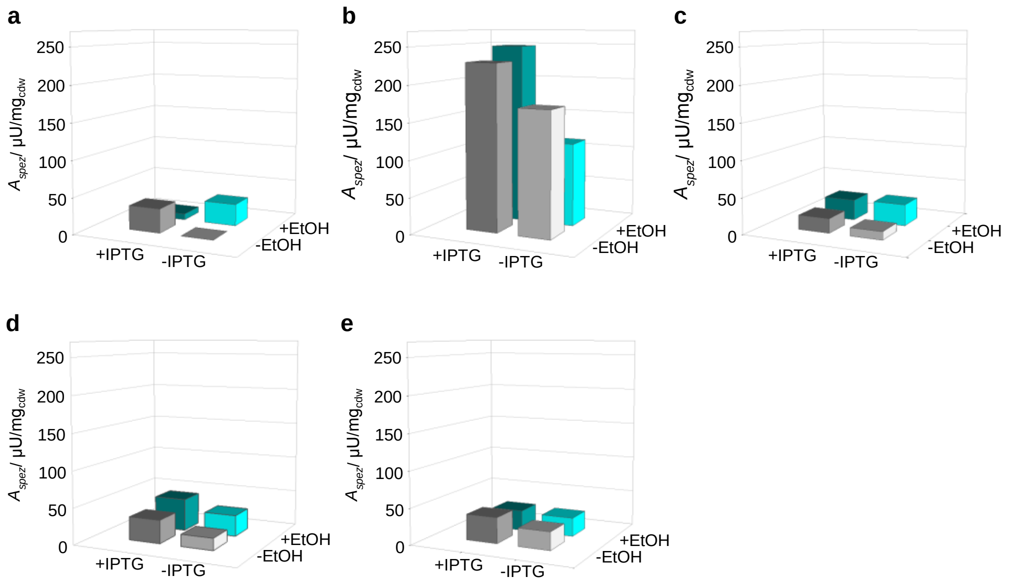

Figure S1 Screening for the cultivation of *E. coli* BL21DE3 coexpressing the recombinant hydantoinase D-Hyd(co) from *A. crystallopoietes* DSM 20117 and different chaperones. **a** C1 **b** C2 **c** C3 **d** C4 **e** C5. Specific activities after whole cell biotransformation with 2 mM PheHyd after cultivation and induction under different conditions. Reactions and measurements were carried out in triplicates, shown are the mean values. Legend: -EtOH +IPTG (*dark grey*), -EtOH -IPTG (*grey*), +EtOH +IPTG (*dark cyan*), +EtOH -IPTG (*cyan*).

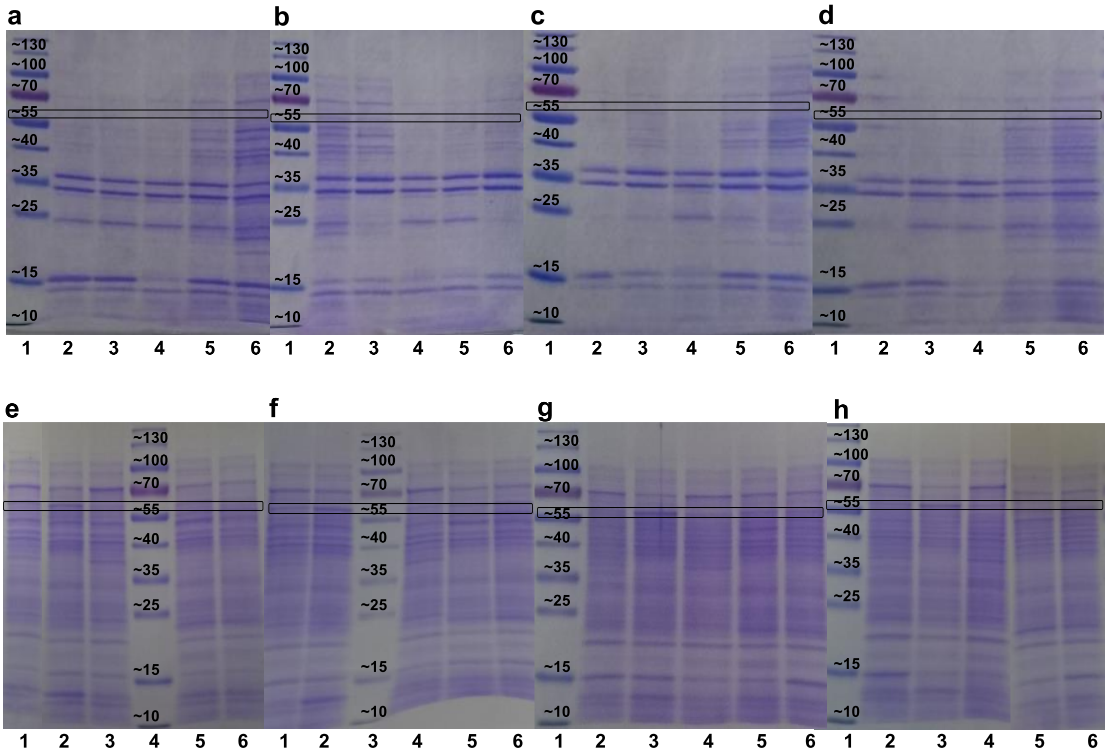

Figure S2 SDS-PAGE of the cultures of *E. coli* BL21DE3 coexpressing the recombinant hydantoinase D-Hyd(co) from *A. crystallopoietes* DSM 20117 and different chaperones after screening for cultivation conditions and subsequent cell disruption. **a** Insoluble fraction EtOH +IPTG **1** protein standard with molecular weights in kDa **2** C1 **3** C2 **4** C3 **5** C4 **6** C5 **b** Insoluble fraction - EtOH -IPTG **1** protein standard with molecular weights in kDa, **2** C1 **3** C2 **4** C3 **5** C4 **6** C5 **c** Insoluble fraction +EtOH +IPTG **1** protein standard with molecular weights in kDa, **2** C1 **3** C2 **4** C3 **5** C4 **6** C5 **d** Insoluble fraction +EtOH -IPTG **1** protein standard with molecular weights in kDa, **2** C1 **3** C2 **4** C3 **5** C4 **6** C5 **e** Soluble fraction -EtOH +IPTG **1** C1 **2** C2 **3** C3 **4** protein standard with molecular weights in kDa **5** C4 **6** C5 **f** Soluble fraction -EtOH -IPTG **1** C1 **2** C2 **3** protein standard with molecular weights in kDa **4** C3 **5** C4 **6** C5 **g** Soluble fraction +EtOH +IPTG **1** protein standard with molecular weights in kDa **2** C1 **3** C2 **4** C3 **5** C4 **6** C5 **h** Soluble fraction +EtOH -IPTG **1** protein standard with molecular weights in kDa **2** C1 **3** C2 **4** C3 **5** C4 **6** C5.
